# Antifungal efficacy of a novel nitric oxide releasing solution in the presence of keratin

**DOI:** 10.3389/fcimb.2026.1753525

**Published:** 2026-04-28

**Authors:** Lisa Long, Ahmed Eltohky, James Sewake, Chris Miller, James Martins, Simon J. L. Teskey, Mahmoud Ghannoum, Thomas S. McCormick

**Affiliations:** 1 Department of Dermatology, Case Western Reserve University, Cleveland, OH, United States; 2 SaNOtize Research and Development Corp, Vancouver, BC, Canada; 3 Department of Dermatology, University Hospitals Cleveland Medical Center, Cleveland, OH, United States

**Keywords:** efinaconazole, keratin, nitric oxide, onychomycosis, terbinafine

## Abstract

**Introduction:**

Nitric oxide (NO)–based therapeutics represent a promising modality for overcoming the challenges of treating dermatophyte infections within keratinized tissues.

**Methods:**

This study evaluated the antifungal activity of a nitric oxide–releasing solution (NORS) against clinical isolates of Trichophyton mentagrophytes, Trichophyton tonsurans, and Epidermophyton floccosum, comparing MIC and MFC profiles to efinaconazole and assessing keratin associated inhibition relative to terbinafine.

**Results:**

NORS demonstrated potent fungicidal activity across all species, with MIC90 and MFC90 values consistently at 0.005 μg/mL. Unlike terbinafine, whose efficacy decreased significantly in keratin-rich environments, NORS retained antifungal activity in the presence of human nail keratin.

**Discussion:**

These results support further clinical evaluation of NO-based antifungals as potential treatments for onychomycosis and tinea pedis.

## 1 Introduction

Onychomycosis and tinea pedis are common superficial fungal infections primarily caused by dermatophytes, a group of keratinophilic fungi that include *Trichophyton rubrum*, *Trichophyton mentagrophytes*, *Trichophyton tonsurans*, and *Epidermophyton floccosum*. These pathogens colonize and degrade keratinized tissues such as the stratum corneum, hair, and nails, resulting in chronic infections that are often difficult to eradicate. Onychomycosis accounts for approximately 50% of all nail disorders and affects up to 14% of the general population, with higher prevalence in elderly and immunocompromised individuals (Elewski, 1998; Gupta et al., 2000; Westerberg and Voyack, 2013). Similarly, tinea pedis, commonly known as athlete’s foot, is one of the most prevalent dermatophytoses worldwide, affecting up to 70% of individuals at some point in their lifetime (Nenoff et al., 2014; Hay and Allen, 2016).

Topical antifungal therapy remains the cornerstone for treating superficial and localized dermatophyte infections, particularly when systemic therapy is contraindicated or not preferred. However, the therapeutic efficacy of topical agents in onychomycosis is often limited by the formidable physical barrier posed by the densely keratinized nail plate (Gupta and Simpson, 2012). To achieve therapeutic concentrations in the infected nail bed, antifungal agents must penetrate the nail plate, reach the site of infection, and retain bioactivity in the keratin-rich environment (Murdan, 2002; Ghannoum et al., 2006). Unfortunately, many antifungal drugs (e.g., terbinafine) exhibit high keratin-binding affinity, which reduces their free drug concentration and fungicidal potential at the site of infection (Tatsumi et al., 2013). Moreover, poor nail permeability and slow nail growth rates contribute to prolonged treatment durations, high recurrence rates, and therapeutic failures (Lipner and Scher, 2019).

Among currently approved topical agents, efinaconazole, a triazole antifungal, has demonstrated superior nail penetration and relatively low keratin-binding properties compared to other topical formulations(9). Nevertheless, treatment failure of antifungals remains common, particularly with *Trichophyton* species that exhibit reduced susceptibility to azoles and allylamines (Singh et al., 2018). Terbinafine (TBF), an allylamine that inhibits squalene epoxidase, is widely used both topically and systemically, but its effectiveness is markedly diminished when used topically in keratinous environments due to drug sequestration and altered pharmacodynamics (Gupta et al., 2014).

Nitric oxide (NO) is an endogenous free radical that plays diverse roles in vascular regulation, neurotransmission, and immune defense (Moncada et al., 1991). In the context of host–pathogen interactions, NO is produced by inducible nitric oxide synthase (iNOS) in host immune cells in response to microbial invasion and has been shown to possess broad-spectrum antimicrobial activity, including antifungal, antibacterial, antiviral, and antiparasitic effects (Fang, 1997; Bogdan, 2001; Shatalin et al., 2011). NO mediates microbial killing through multiple mechanisms, including nitrosative stress, DNA damage, protein oxidation, lipid peroxidation, and disruption of microbial biofilms (De Groote and Fang, 1995; Wink et al., 2011). Notably, NO has been reported to inhibit fungal growth and germination, delay mycelial extension, and reduce fungal burden in both *in vitro* and *in vivo* models (Mozaffarian et al., 1997; Regev‐Shoshani et al., 2013).

The potential utility of nitric oxide-releasing formulations in dermatology has gained interest due to their multitargeted mechanism of action, minimal risk of developing resistance, and ability to penetrate biofilms and keratinized barriers (Friedman et al., 2008; Martinez et al., 2009). Recent advances have enabled the development of stabilized nitric oxide-releasing solutions and gels (NORS/NORS-gel), which allow for controlled, sustained delivery of NO at therapeutic concentrations directly to infected tissues. Preliminary studies suggest that NORS may be effective against a broad spectrum of cutaneous pathogens, including dermatophytes, and may offer advantages over conventional antifungal therapies in treating recalcitrant infections such as onychomycosis (Friedman et al., 2008; Stasko et al., 2018).

The present study was conducted to evaluate the antifungal activity of a proprietary nitric oxide-releasing solution (NORS) against a panel of susceptible and resistant dermatophyte isolates, and to compare its activity with that of efinaconazole. In addition, the influence of keratin on antifungal performance was compared between NORS and TBF in the presence of human nail keratin. By addressing both fungicidal potency and keratin-binding limitations, this investigation aims to explore the therapeutic potential of nitric oxide-based topical treatments in the management of superficial mycoses.

## 2 Methods

### 2.1 Materials

Nails from the great toe of human cadavers were purchased from Science Care, AZ, USA, and stored at −80 °C until use.

### 2.2 Antifungals

The nitric oxide releasing solution was prepared by SaNOtize/NoWonder according to a proprietary formulation. Efinaconazole and TBF were purchased from Sigma Aldrich (Saint Louis, MO).

### 2.3 Fungal strains

The clinical strains of *Trichophyton tonsurans*, *Trichophyton mentagrophytes*, and *Epidermophyton floccosum* (*organism MRLs listed in **Tables 1**, **2**) used for the susceptibility testing assays were provided by the Center for Medical Mycology, Department of Dermatology at Case Western Reserve University, and University Hospitals Cleveland Medical Center, Cleveland, OH, and were derived from clinical specimens as indicated in **Table 1**. The present work focused on *T. mentagrophytes*, *T. tonsurans*, and *Epidermophyton floccosum* due to the availability of well-characterized clinical isolates and their relevance in emerging antifungal resistance patterns. *Trichophyton mentagrophytes* (ATCC 37035) used for the keratin assays was purchased from the American Type Culture Collection (ATCC, Manassas, VA, USA). Reference strains were kept on Potato Dextrose Agar (PDA) slants and stored at −80 °C until use. Fungal cultures were streaked on Sabouraud dextrose agar and incubated at 25 °C for 7 days before being sealed with parafilm and stored at 4 °C. Fungal cultures were streaked every 28 days to ensure viable cells.

**Table 1:** Minimum inhibitory concentrations (MIC) of NORS compared to EFI against dermatophyte clinical isolates.

MIC (µg/mL)				
Organism	Isolate ID	Source	NORS	Efinaconazole
*E. floccosum*	10776	Right toenail	0.003	≤0.004
*E. floccosum*	43153	Hair/scalp	0.005	≤0.004
*E. floccosum*	24909	Skin—foot	0.005	0.125
*E. floccosum*	24911	Skin—foot	0.002	0.125
*E. floccosum*	25447	Left toenail	0.005	0.125
*E. floccosum*	25545	Skin—foot	0.005	0.125
*E. floccosum*	26712	Skin—foot	0.005	0.125
*E. floccosum*	27573	Skin—foot	0.001	0.25
*E. floccosum*	43279	Toenail	0.003	0.25
*E. floccosum*	43363	Skin—foot	0.005	0.25
*T. mentagrophytes*	42831	Skin—body	0.005	≤0.004
*T. mentagrophytes*	43110	Toenail	0.005	≤0.004
*T. mentagrophytes*	42829	Skin—groin	0.005	0.002
*T. mentagrophytes*	42315	Skin—body	0.005	0.002
*T. mentagrophytes*	42572	Skin—foot	0.005	0.031
*T. mentagrophytes*	4439	Toenail	0.005	0.016
*T. mentagrophytes*	43220	Toenail	0.005	0.016
*T. mentagrophytes*	43395	Skin—body	0.005	0.016
*T. mentagrophytes*	42572	Skin—foot	0.005	0.031
*T. mentagrophytes*	42323	Toenail	0.005	0.06
*T. mentagrophytes*	37067	Isolate	0.005	0.25
*T. tonsurans*	37067	Isolate	0.005	0.25
*T. tonsurans*	43032	Hair/scalp	0.005	≤0.004
*T. tonsurans*	43360	Hair/scalp	0.005	≤0.004
*T. tonsurans*	43403	Hair/scalp	0.005	≤0.004
*T. tonsurans*	43501	Toenail	0.005	≤0.004
*T. tonsurans*	43271	Hair/scalp	0.005	0.002
*T. tonsurans*	43256	Hair/scalp	0.005	0.004
*T. tonsurans*	35697	Isolate	0.005	0.125
*T. tonsurans*	36813	Isolate	0.005	0.25
*T. tonsurans*	37275	Isolate	0.003	0.25
*T. tonsurans*	43480	Hair/scalp	0.005	0.25

**Table 2:** Minimum fungicidal concentrations (MFC) of NORS compared to EFI against selected dermatophyte clinical isolates.

MFC			
Organism	Isolate ID	NORS (dilution×)	Efinaconazole (µg/ml)
*E. floccosum*	10776	0.005	0.008
*E. floccosum*	43153	0.005	0.008
*E. floccosum*	24909	0.005	0.5
*E. floccosum*	24911	0.005	0.5
*E. floccosum*	25545	0.005	0.5
*E. floccosum*	43279	0.005	0.5
*E. floccosum*	25447	0.005	>0.5
*E. floccosum*	26712	0.005	>0.5
*E. floccosum*	27573	0.003	>0.5
*E. floccosum*	43363	0.005	>0.5
*T. mentagrophytes*	43395	0.005	0.06
*T. mentagrophytes*	42831	0.005	0.125
*T. mentagrophytes*	4439	0.005	0.25
*T. mentagrophytes*	42315	>0.005	0.5
*T. mentagrophytes*	43110	0.005	0.5
*T. mentagrophytes*	43220	0.005	0.5
*T. mentagrophytes*	37067	0.005	>0.5
*T. mentagrophytes*	42572	0.005	>0.5
*T. mentagrophytes*	42829	0.005	>0.5
*T. mentagrophytes*	42323	0.005	>0.5
*T. tonsurans*	43360	0.005	0.016
*T. tonsurans*	43032	0.005	0.03
*T. tonsurans*	43403	0.005	0.03
*T. tonsurans*	35697	0.005	0.5
*T. tonsurans*	37275	0.005	0.5
*T. tonsurans*	43501	0.005	>0.5
*T. tonsurans*	36813	0.005	>0.5
*T. tonsurans*	43256	0.005	>0.5
*T. tonsurans*	43271	0.005	>0.5
*T. tonsurans*	43480	0.005	>0.5

### 2.4 Susceptibility testing assays

Minimal inhibitory concentration (MIC) and minimal fungicidal concentration (MFC) were performed comparing NORS to efinaconazole against 10 strains each of *Trichophyton tonsurans*, *T. mentagrophytes*, and *Epidermophyton floccosum.*

To determine MIC values, fungal suspensions in potato dextrose broth were created at a concentration of 1×10^4^ conidia/mL and 180 µL of fungal suspension per well was added to a 96-well plate. NORS was prepared within 30 min of testing as follows: Components A and B were mixed in a 1:1 ratio in a centrifuge tube to form the activated compound (10× NORS, initial concentration ~ 58.2 μg/mL). Serial dilutions were performed in acidified saline to obtain the different dilutions of NORS as tested. Efinaconazole was suspended in dimethyl sulfoxide. Next, 20µL of NORS or efinaconazole solutions with steadily decreasing concentrations were added to each of the wells. The plates were incubated at 25 °C for 7 days, and growth inhibition was evaluated.

The MFC determination was performed according to the method previously described by Ghannoum and Isham (Isham and Ghannoum, 2007). Specifically, the total contents of each clear well from the MIC assay was subcultured onto PDA. To avoid antifungal carryover, the aliquots were allowed to soak into the agar and then streaked for isolation once dry, thus removing the cells from the drug source. Petri dishes were incubated at 35 °C for 48 h and the number of colony-forming units (CFUs) determined. All MIC and MFC determinations were performed in triplicate as independent experiments. Susceptibility testing was performed using a broth microdilution method consistent with the CLSI M38-Reference Method for Broth Dilution Antifungal Susceptibility Testing of Filamentous Fungi (CLSI, 2017).

### 2.5 Keratin assays

To prepare keratin, human cadaver nails were ground to powder and sterilized. A saline suspension of *T. mentagrophytes* (ATCC 37035, TBF MIC value of 0.002 µg/mL) was prepared to a concentration of 1×10^7^ conidia/mL. 1-mL aliquots of conidial suspension were added to the following combinations: (1) NORS (~58.2 µg/mL), (2) NORS (~58.2 µg/mL) + keratin (50 mg/mL), (3) TBF (0.002 µg/mL), (4) TBF (0.002 µg/mL) + keratin (50 mg/mL), (5) control (*T. mentagrophytes* in saline only), (6) control + keratin (50 mg/mL). Following 15 min of exposure, tubes were centrifuged, the supernatant decanted, and the cell pellets resuspended in 1 mL sterile saline. A 15-min exposure time was selected to evaluate early fungicidal activity, consistent with the rapid antimicrobial kinetics of nitric oxide–based compounds. Next, 10-fold serial dilutions of the prepared solutions were plated on PDA to determine the number of CFUs. This procedure was repeated in triplicate.

## 3 Results

### 3.1 Antifungal effect of NORS

The range, MIC_50_, and MIC_90_ for NORS against the *E. floccosum* isolates were 0.001-0.005, 0.005, and 0.005 µg/mL, respectively. For efinaconazole (EFI), the range, MIC_50_, and MIC_90_ were ≤0.004-0.25, 0.125, and 0.25 µg/mL, respectively. The range, MIC_50_, and MIC_90_ for NORS against the *T. mentagrophytes* isolates tested were 0.005->0.005, 0.005, and 0.005 µg/mL, respectively. For EFI, the range, MIC_50_, and MIC_90_ were ≤0.004-0.25, 0.016, and 0.06 µg/mL, respectively, while the range, MIC_50_, and MIC_90_ for NORS against the *T. tonsurans* isolates were 0.003->0.005, 0.005, and 0.005 µg/mL, respectively. For EFI, the range, MIC_50_, and MIC_90_ were ≤0.004-0.25, 0.002, and 0.25 µg/mL, respectively. Against all the dermatophytes tested, the range, MIC_50_, and MIC_90_ for NORS against all isolates were 0.003->0.005, 0.005, and 0.005 µg/mL, respectively. For EFI, the range, MIC_50_, and MIC_90_ were ≤0.004-0.25, 0.016, and 0.25 µg/mL, respectively (**Table 3**).

**Table 3:** MIC range, MIC_50_, and MIC_90_ for NORS and EFI against the dermatophytes tested (n=30).

Parameter	*T. mentagrophytes* (n=10)		*T. tonsurans* (n=10)		*E. floccosum* (n=10)		All dermatophytes (n=30)	
	NORS (µg/mL)	EFI (µg/mL)	NORS (µg/mL)	EFI (µg/mL)	NORS (µg/mL)	EFI (µg/mL)	NORS (µg/mL)	EFI (µg/mL)
Range	0.005->0.005	≤0.004-0.25	0.003-0.005	≤0.004-0.25	0.001-0.005	≤0.004-0.25	0.003->0.005	≤0.004-0.25
MIC_50_	0.005	0.016	0.005	0.002	0.005	0.125	0.005	0.016
MIC_90_	0.005	0.06	0.005	0.25	0.005	0.25	0.005	0.25

The range, MFC_50_, and MFC_90_ for NORS against the *E. floccosum* isolates were 0.003-0.005, 0.005, and 0.005 µg/mL, respectively. For EFI, the range, MFC_50_, and MFC_90_ were 0.008->0.5, >0.5, and >0.5 µg/mL, respectively. The range, MFC_50_, and MFC_90_ for NORS against the *T. mentagrophytes* isolates tested were 0.005->0.005, 0.005, and 0.005 µg/mL, respectively. For EFI, the range, MFC_50_, and MFC_90_ were 0.06->0.5, 0.5, and >0.5 µg/mL, respectively. The range, MFC_50_, and MFC_90_ for NORS against the *T. tonsurans* isolates were 0.005->0.005, 0.005, and 0.005 µg/mL, respectively. For EFI, the range, MFC_50_, and MFC_90_ were 0.16->0.5, >0.5, and >0.5 µg/mL, respectively. Furthermore, the range, MFC_50_, and MFC_90_ for NORS were 0.003->0.005, 0.005, and 0.005 µg/mL, respectively. For EFI, the range, MFC_50_, and MFC_90_ were ≤0.008->0.5, 0.5, and >0.5 µg/mL, respectively (**Table 4**).

**Table 4:** MFC range, MFC_50_, and MFC_90_ for NORS and EFI against the dermatophytes tested (n=30).

Parameter	*T. mentagrophytes* (n=10)		*T. tonsurans* (n=10)		*E. floccosum* (n=10)		All dermatophytes (n=30)	
	NORS (µg/mL)	EFI (µg/mL)	NORS (µg/mL)	EFI (µg/mL)	NORS (µg/mL)	EFI (µg/mL)	NORS (µg/mL)	EFI (µg/mL)
Range	0.005->0.005	0.06->0.5	0.005->0.005	0.16->0.5	0.003-0.005	0.008->0.5	0.003->0.005	≤0.008->0.5
MFC_50_	0.005	0.5	0.005	>0.5	0.005	0.5	0.005	>0.5
MFC_90_	0.005	>0.5	0.005	>0.5	0.005	>0.5	0.005	>0.5

### 3.2 NORS activity in the presence of keratin

**Figure 1:**
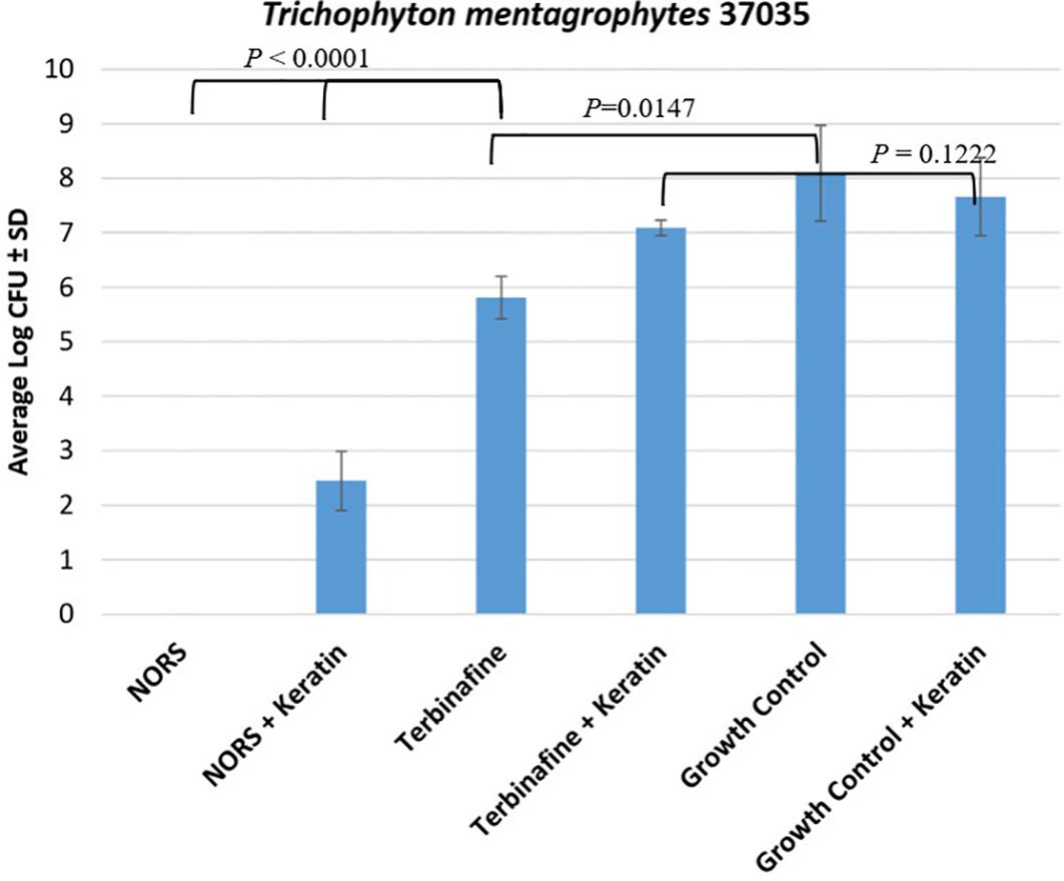
Average log colony forming units (CFUs)/mL ± SD for the tested compounds (NORS and TBF) against *T. mentagrophytes* (37035) in the presence and absence of keratin.

Keratin inhibition assays were conducted using *T. mentagrophytes* (ATCC 37035) in the presence and absence of powdered human nail keratin to evaluate the effect of keratin binding on antifungal activity. Colony-forming units (CFUs) were enumerated following treatment with NORS and terbinafine. NORS without and with keratin demonstrated the lowest fungal burdens with average log CFUs/mL ± SD of 0 ± 0 and 2.45 ± 0.5, respectively. In contrast, the TBF-treated samples without and with keratin showed average log CFUs/mL ± SD of 5.81 ± 0.4 and 7.09 ± 0.1, respectively (**Figure 1**).

### 3.3 Keratin assay

**Figure 1**. NORS without and with keratin demonstrated the lowest fungal burdens with average log CFUs/mL ± SD of 0 ± 0 and 2.45 ± 0.5, respectively. In contrast, the TBF-treated samples without and with keratin showed average log CFUs/mL ± SD of 5.81 ± 0.4 and 7.09 ± 0.1, respectively. Moreover, NORS in the absence and presence of keratin demonstrated significant activity when compared to both the growth control and TBF (*P* < 0.0001). However, TBF alone significantly reduced CFUs compared to the growth control (*P* = 0.0147). In contrast, TBF with keratin did not significantly reduce CFUs compared to the growth control (*P* = 0.1222).

## 4 Discussion

The present study demonstrates that NORS possesses potent antifungal activity against multiple dermatophyte species, including *Trichophyton tonsurans*, *Trichophyton mentagrophytes*, and *Epidermophyton floccosum*. NORS exhibited inhibitory and fungicidal properties that were comparable or superior to those of efinaconazole, a topical triazole currently approved for the treatment of onychomycosis. Notably, the efficacy of NORS extended to isolates with relatively higher MIC to efinaconazole, suggesting that NORS may be effective against strains with elevated MIC to conventional antifungals (Vitiello et al., 2023). However, it will be interesting to conduct susceptibility testing of NORS against isolates that are known to be resistant to other antifungals.

One of the most significant challenges in treating onychomycosis is the ability of antifungal agents to penetrate the dense, keratinized nail plate and maintain therapeutic activity within the nail bed. Keratin binding has been shown to limit the bioavailability of several topical agents, notably TBF, whose antifungal efficacy is substantially reduced in keratin-rich environments (Sugiura et al., 2014). In this study, keratin binding assays revealed that NORS retained antifungal activity in the presence of human nail keratin, whereas TBF’s activity was significantly attenuated under similar conditions. This finding supports the hypothesis that NO-based therapies may offer a unique advantage compared to TBF by circumventing keratin-associated therapeutic barriers (Stasko et al., 2018). In the current study, a 15-min exposure time was used to assess early fungicidal activity; consistent with the rapid antimicrobial kinetics of nitric oxide, follow-up studies will be necessary that extend exposure time in order to assess sustained antifungal activity.

The mechanisms underlying the antifungal efficacy of nitric oxide are likely multifactorial. NO exerts cytotoxic effects via nitrosative and oxidative stress, damaging fungal DNA, proteins, and cell membranes, while also disrupting cellular respiration and metabolic pathways (Brown et al., 2009; Grayton et al., 2024). Unlike traditional antifungals, NO does not target ergosterol synthesis (the main target for the azoles) or fungal cell wall enzymes (ex. β-1,3-D-glucan synthase) which the echinocandins target, thereby potentially reducing the risk of cross-resistance. The lack of significant keratin binding and its ability to diffuse through biofilms further enhance its therapeutic profile for superficial and subungual infections (Costa-Orlandi et al., 2021).

## 5 Limitations of the current study

This study has several strengths, including the use of multiple dermatophyte species, assessment of both MIC and MFC values, and direct comparison with established antifungals. Limitations of the current study include its *in vitro* nature with several caveats based on this approach. The limited variability observed in MIC and MFC values for NORS may reflect its multitarget mechanism of action. However, additional studies using expanded concentration ranges and repeated testing are warranted to confirm these findings. While cadaveric human nail and standardized fungal strains were employed to simulate clinical conditions, *in vivo* studies are necessary to confirm the pharmacokinetics, tolerability, and therapeutic efficacy of NORS in patients with dermatophytosis. Additional limitations include the absence of *Trichophyton rubrum*, the most prevalent cause of onychomycosis. Future studies will incorporate this species to further establish clinical applicability. The current study did not include cytotoxicity or biocompatibility assessments. While nitric oxide–releasing formulations have demonstrated acceptable safety profiles in prior studies, dedicated cytotoxicity evaluation in human skin models will be required to support clinical translation. Finally, the keratin interference assay was performed using a single *T. mentagrophytes* reference strain. While sufficient for proof of concept, this limits generalizability across dermatophyte species.”

## 6 Conclusion

NORS demonstrated broad-spectrum antifungal and fungicidal activity against susceptible and strains with relatively higher MIC to dermatophytes. Importantly, NORS demonstrated a statistically significant efficacy in the presence of keratin, in contrast to terbinafine which failed to do so. These findings support the potential of nitric oxide-based therapies as a novel approach to overcome the therapeutic limitations associated with conventional topical antifungals, including treatment resistance, in the management of onychomycosis and tinea pedis. Further clinical studies are warranted to validate these results and assess long-term safety and efficacy in real-world patient populations.

## Data Availability

The raw data supporting the conclusions of this article will be made available by the authors, without undue reservation.
